# ASS234, As a New Multi-Target Directed Propargylamine for Alzheimer's Disease Therapy

**DOI:** 10.3389/fnins.2016.00294

**Published:** 2016-06-28

**Authors:** José Marco-Contelles, Mercedes Unzeta, Irene Bolea, Gerard Esteban, Rona R. Ramsay, Alejandro Romero, Ricard Martínez-Murillo, M. Carmo Carreiras, Lhassane Ismaili

**Affiliations:** ^1^Laboratory of Medicinal Chemistry, Institute of General Organic Chemistry, Cajal Institute (CSIC)Madrid, Spain; ^2^Departament de Bioquímica i Biologia Molecular, Facultat de Medicina, Institut de Neurociències, Universitat Autònoma de BarcelonaBarcelona, Spain; ^3^Biomedical Sciences Research Complex, University of St AndrewsSt Andrews, UK; ^4^Department of Toxicology and Pharmacology, Faculty of Veterinary Medicine, Complutense University of MadridMadrid, Spain; ^5^Neurovascular Research Group, Department of Molecular, Cellular and Developmental Neurobiology, Cajal Institute (CSIC)Madrid, Spain; ^6^Research Institute for Medicines and Pharmaceutical Sciences (iMed.ULisboa), Faculty of Pharmacy, University of LisbonLisbon, Portugal; ^7^Laboratoire de Chimie Organique et Thérapeutique, Neurosciences Intégratives et Cliniques EA 481, Université Franche-Comté, Université Bourgogne Franche-Comté, UFR SMPBesançon, France

**Keywords:** multi-target directed ligands, Alzheimer's disease, monoamine oxidases, cholinesterases, drugs

## Abstract

**Highlights:**

- **ASS2324** is a hybrid compound resulting from the juxtaposition of donepezil and the propargylamine **PF9601N**
- **ASS2324** is a multi-target directed propargylamine able to bind to all the AChE/BuChE and MAO A/B enzymes
- **ASS2324** shows antioxidant, neuroprotective and suitable permeability properties
- **ASS2324** restores the scopolamine-induced cognitive impairment to the same extent as donepezil, and is less toxic
- **ASS2324** prevents β-amyloid induced aggregation in the cortex of double transgenic mice
- **ASS2324** is the most advanced anti-Alzheimer agent for pre-clinical studies that we have identified in our laboratories

The complex nature of Alzheimer's disease (AD) has prompted the design of Multi-Target-Directed Ligands (MTDL) able to bind to diverse biochemical targets involved in the progress and development of the disease. In this context, we have designed a number of MTD propargylamines (MTDP) showing antioxidant, anti-beta-amyloid, anti-inflammatory, as well as cholinesterase and monoamine oxidase (MAO) inhibition capacities. Here, we describe these properties in the MTDL **ASS234**, our lead-compound ready to enter in pre-clinical studies for AD, as a new multipotent, permeable cholinesterase/monoamine oxidase inhibitor, able to inhibit Aβ-aggregation, and possessing antioxidant and neuroprotective properties.

## Introduction

Alzheimer's disease (AD) is the most common neurodegenerative disease in the elderly (Karlawish, 2011). AD is characterized by progressive neuronal death resulting in severe cognitive impairment. Two distinctive hallmarks of AD are the presence of accumulated beta-amyloid (Aβ) plaques (Hamley, 2012) and hyperphosphorylated tau protein in the form of intracellular neurofibrillary tangles (NFT) (Wang et al., 2013). Although the precise etiology of AD is not yet known, there is a large consensus in describing it as a complex disorder caused by many factors, including loss of cholinergic transmission, protein misfolding and Aβ aggregation, oxidative stress, free radical formation (Rosini et al., 2014), and metal dyshomeostasis (Huang et al., 2004). AD pathology also involves dysfunctional neurotransmittters and synapse loss (Aisa et al., 2010; Villemagne and Chételat, 2016).

In the following sections we describe the currently accepted AD hypotheses, and our recent contribution to the development of new multipotent propargylamines for AD therapy.

## Alzheimer's disease hypotheses

### Cholinergic hypothesis

Cholinergic neurotransmission modulates both cognitive function and cortical plasticity (Arendt and Bigl, 1986) and plays a significant role in the control of cerebral blood flow (Biesold et al., 1989), cortical activity (Détári et al., 1999), learning and memory (Deutsch, 1971). The first physiological evidence of the involvement of the cholinergic system in AD pathology was a reduction of the neurotransmitter acetylcholine (ACh), which constitutes the basis of the cholinergic hypothesis of AD (Deutsch, 1971), used to discover the first anti-AD agents. Acetylcholinesterase (AChE) is expressed in cholinergic neurons, its primary function being the rapid breakdown of ACh during the cholinergic neurotransmission. In addition to rapid breakdown by AChE, ACh can also be metabolized by butyrylcholinesterase (BuChE) (Mesulam et al., 2002) but with different kinetic behavior. Whereas AChE pre-dominates in neurons and exhibits high affinity for ACh, BuChE is present in endothelia, glia and neuronal cells with low affinity for ACh and high *K*_M_ values (Soreq and Seidman, 2001).

### β-amyloid cascade hypothesis

The amyloid hypothesis postulates that neurodegeneration in AD is caused by abnormal accumulation of Aβ plaques in various areas of the brain (Evin and Weidemann, 2002). The Aβ senile plaques contain Aβ peptides with 39–43 amino acid residues, proteolytically derived from the sequential enzymatic action of β- and γ-secretases of transmembrane APP (Coulson et al., 2000). Within plaques, Aβ peptides in β-sheet conformation assemble and polymerise into fibrillar, protofibers and polymorphic oligomers (Selkoe, 1994). *In vitro*, the Aβ aggregation process is highly susceptible to pH, ionic strength of the solvent, purification process and temperature. Distinct oligomerization and assembly processes between Aβ_1−40_ and Aβ_1−42_ have been described (Bitan et al., 2003). While dimers and trimers are the most toxic forms of Aβ_1−42_, Aβ_1−40_ reaches equilibrium from monomers to tetramers. Recent findings have shown that soluble oligomeric species were able to disrupt synaptic function (Lambert et al., 1998) and support the belief that soluble dimeric species are highly toxic (Jin et al., 2011). However, direct Aβ-peptide neurotoxicity has been difficult to prove in animal models (Serrano-Pozo et al., 2013). Since the postulation of the amyloid hypothesis, a number, but a number of unsuccessful efforts have been undertaken in clinical research in order to develop novel drugs based on this concept.

### Oxidative stress

Increased production of Reactive Oxygen Species (ROS) have been observed in AD (Praticò, 2008) and, consequently, elevated levels of oxidative markers including damage to proteins, lipids, carbohydrates, and nucleic acids. Antioxidant enzymes were also found to be increased in specific AD brain regions (Sultana et al., 2011). Not surprisingly, the oxidative stress (OS) hypothesis of AD has emerged as a key event in the progress of the disease. In addition, evidence suggests that secretion and deposition of Aβ within the neurons are compensatory measures taken by cells in effort to protect themselves against damage triggered by OS (Hayashi et al., 2007).

Cellular oxidative damage has also been linked to tau hyperphosphorylation and formation of NFTs (Lee et al., 2004). As a consequence, cells succumb to neurodegeneration exhibiting the distinctive cognitive impairment observed in AD patients (Zhu et al., 2007). Altogether, the primary role of OS in AD has been overwhelmingly confirmed, offering the chance to develop specific disease-modifying antioxidant approaches to cure or prevent the development of the disease.

### Biometal hypothesis

The increased levels of ROS are reflected in a deregulated content of biometals such as iron, copper and zinc in the brain of AD patients. Recent findings point to brain OS as one of the earliest changes in AD pathogenesis that might play a central role in the disease progression (Lee et al., 2010). Redox-active metals are capable of stimulating free radical formation *via* the Fenton reaction. Biometals have also been shown to mediate Aβ toxicity in AD (Duce et al., 2010). It has been shown that Aβ peptide itself is a strong redox-active metalloprotein able to directly produce hydrogen peroxide and OH^−^ in the presence of copper or iron, which, in turn, are enriched in the amyloid cores of senile plaques (Huang et al., 1999). Also, biometals can interact directly with Aβ peptide enhancing its self-aggregation and oligomerization at low physiological concentrations or at mildly acidic conditions (Huang et al., 1999). Moreover, metals can promote tau hyperphosphorylation and subsequent formation of NFTs inducing its aggregation upon interaction with Aβ (Yamamoto et al., 2002).

## Drugs for AD therapy

To date, only five drugs have ever been approved for AD therapy. Tacrine, rivastigmine, galantamine and donepezil are AChEI, whereas memantine is a NMDA receptor antagonist.

Tacrine, a competitive AChEI and the first drug to be approved for use in AD by the FDA in 1993, was withdrawn from the market in 2013 due to the high incidence of side effects, mostly derived from hepatotoxicity (Qizilbash et al., 1998). Rivastigmine, a non-selective pseudoreversible ChE inhibitor (Bullock and Lane, 2007), has been reported to have less side effects as well as positive benefit after administration to mild-to-moderate AD patients (Birks and Grimley Evans, 2015). Galantamine, a weak competitive reversible AChEI (Greenblatt et al., 1999) is also a potent allosteric modulator of nicotinic acetylcholine receptors—α_4_β_2_, α_7_/5-HT_3_, α_3_β_4_, and α_6_β_4_—in certain areas of the brain, and potentiates the effects of orthoesteric agonists (Dajas-Bailador et al., 2003; Akk and Steinbach, 2005). Donepezil is a brain-permeable reversible non-competitive ChE inhibitor approved for use in AD (Birks and Harvey, 2006) and currently the most widely prescribed drug for the treatment of this disease. Donepezil is highly selective for AChE over BuChE activity (405:1) (Nochi et al., 1995). Compared to other approved AChEI, donepezil is similarly effective in ameliorating cognitive and functional decline in AD with comparable safety and tolerability (Doody et al., 2014).

Memantine is a glutamatergic agent, the first and only NMDA receptor antagonist approved by FDA in 2003 for the treatment of moderate-to-severe AD and dementia. Memantine binds to NMDA receptors with a low-micromolar IC_50_ value, exhibits neuroprotective activities against Aβ toxicity (Hu et al., 2007), tau phosphorylation (Song et al., 2008), neuroinflammation (Willard et al., 2000), and oxidative stress (Figueiredo et al., 2013).

In the face of general neuronal loss, monoamine oxidase (MAO) inhibitors are used to preserve remaining levels of catecholamine neurotransmitters by inhibiting MAO A in neurons or MAO B in serotonergic neurons, glia and astrocytes. Since MAO B activity is increased in AD, MAO B inhibitors may be of potential therapeutic interest both to maintain neurotransmitter levels and to decrease hydrogen peroxide production (Mandel et al., 2005). For example, rasagiline and selegiline are propargylamines that irreversibly inhibit brain MAO B, but also show neuroprotective activities mainly due to their propargyl moiety (Zindo et al., 2015).

The lack of therapeutic effectiveness of the current drugs based on the single-target paradigm (León et al., 2013) for the treatment of AD prompted the search of MTDL, designed by molecular hybridization of different pharmacophoric moieties from well-known bioactive molecules, able to bind to multiple targets associated with AD. As a result, a number of standard natural or synthetic compounds, including donepezil, tacrine or rivastigmine (Samadi et al., 2011), curcumin (Malar and Devi, 2014), berberine (Jiang et al., 2011) or 8-hydroxyquinoline (Gomes et al., 2014) have been used for this purpose.

**Figure 1:**
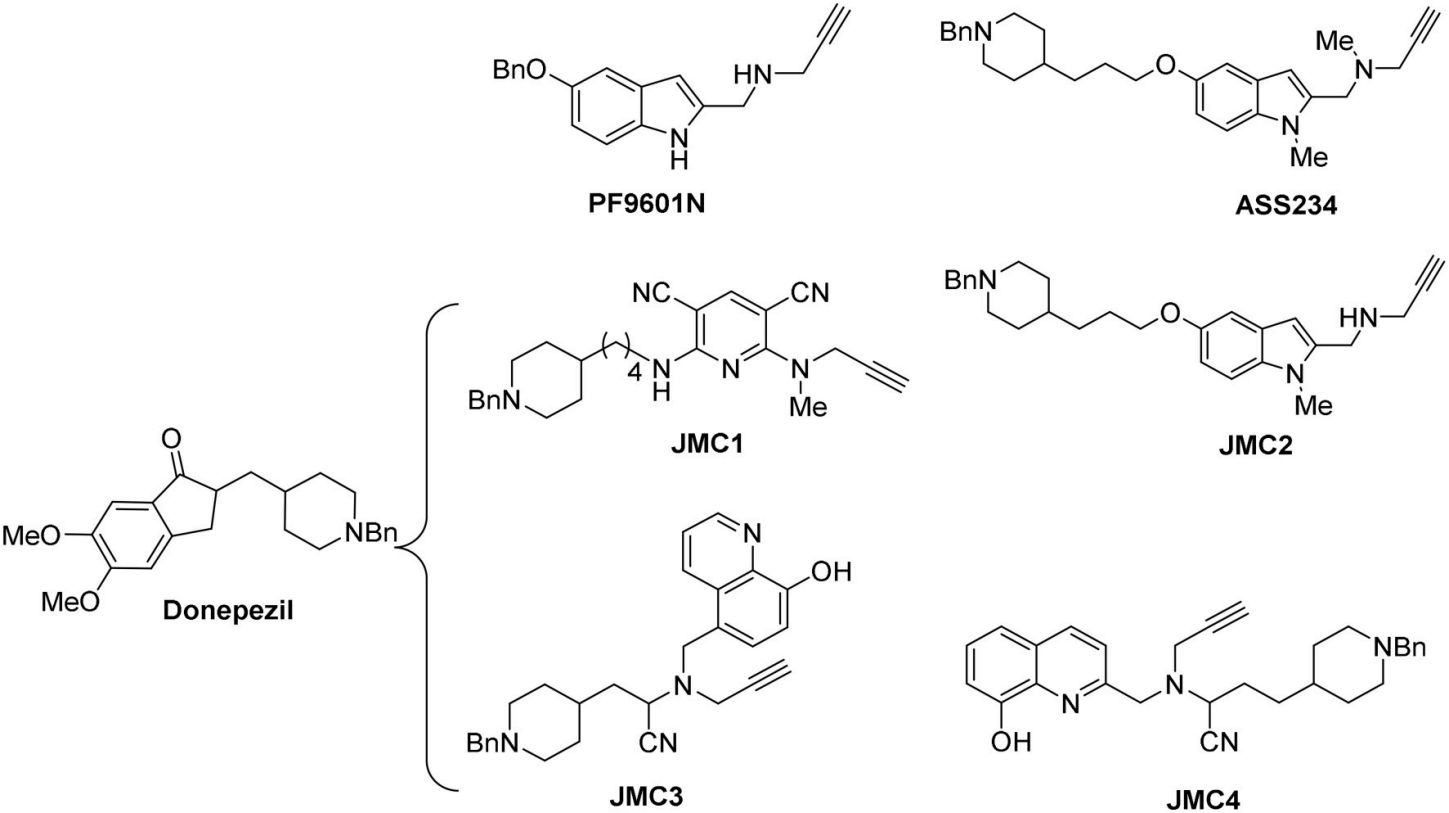
**Structure of compounds donepezil, PF9601N, ASS234, and JMC1-4**.

Based on this background, we have designed several MTD propargylamines (MTDP) for the potential treatment of AD. All these compounds bear the *N*-benzylpiperidine group present in donepezil and the *N*-propargylamine motif present in L-deprenyl (used in Parkinson's disease) and in **PF9601N** (Figure 1), a potent and selective MAO B inhibitor with neuroprotective effect demonstrated *in vitro* and *in vivo* using different experimental models (Pérez et al., 1999). The donepezil motif gives inhibition of the cholinesterases as well as the ability to inhibit the aggregation of Aβ via the acetylcholinesterase peripheral site, whereas the propargylamine inhibits MAO enzymes and also gives neuroprotection. Both scaffolds were linked by different heterocyclic ring systems, such as pyridine, indole or 8-hydroxyquinoline, affording diverse MTDP as promising drugs to be used in AD therapy (Figure 1). This strategy led to **ASS234** (Figure 1) with the anti-cholinergic activity of donepezil, selective MAO A inhibition and neuroprotective properties (Bolea et al., 2011).

Next, with the aim of searching for improved MTDLs, two series of novel structurally derived compounds from **ASS234** as multipotent donepezil-pyridyl and donepezil-indolyl hybrids were designed and pharmacologically assessed. Thus, the donepezil-pyridyl compound **JMC1** (Figure 1) was identified as a very potent hAChE inhibitor (IC_50_ = 1.1 nM) and a moderate hBuChE inhibitor (IC_50_ = 0.6 μM) with total selectivity toward human MAO B (hMAO B) (Bautista-Aguilera et al., 2014b). The donepezil-indole **JMC2** (Figure 1) exhibited the most interesting profile as a potent MAO A inhibitor (IC_50_ = 5.5 nM) moderately able to inhibit MAO B (IC_50_ = 150 nM), AChE (IC_50_ = 190 nM), and BuChE (IC_50_ = 830 nM) (Bautista-Aguilera et al., 2014a). Moreover, the kinetic analysis showed that **JMC2** is a mixed-type AChE inhibitor able to span both the catalytic and peripheral sites (CAS and PAS) of this enzyme, a fact further confirmed by molecular modeling studies. Propargylamines **JMC3** (Wang et al., 2014) and **JMC4** (Wu et al., 2015) bear a *N*-benzylpiperidine moiety from donepezil and a 8-hydroxyquinoline group (Figure 1). **JMC3** (Figure 1) was further characterized as an irreversible MAO and mixed-type ChE inhibitor in low micromolar range, and, in addition, it strongly complexed Cu (II), Zn (II), and Fe (III) (Wang et al., 2014). From theoretical ADMET analyses, **JMC3** exhibited proper drug-likeness properties and good brain penetration suitable for CNS activity. **JMC4** (Figure 1) showed similar inhibitory behavior as dual ChE/MAO inhibitor (Wu et al., 2015).

**Figure 2:**
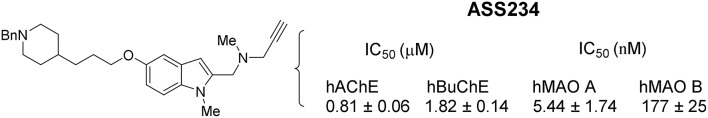
**Structure and IC_50_ values for the inhibition of ChEs and MAO enzymes by ASS234**.

The propargylamine **ASS234** (Figure 1), deserves further discussion. **ASS234** is a very potent human MAO A and MAO B inhibitor with a IC_50_ values of 5.44 ± 1.74 and 177 ± 25 nM, respectively, inhibiting also both ChEs (IC_50_ (human AChE) = 0.81 ± 0.06 μM; IC_50_ (human BuChE) = 1.82 ± 0.14 μM) (Esteban et al., 2014) (Figure 2). In contrast, the reference compounds had only their single expected activity: donepezil was ineffective at inhibiting MAO activities, and **PF9601N**, while potently and selectively inhibiting MAO B displayed no interaction with the ChEs (Bolea et al., 2011). To sum up, **ASS234** combines the best properties of donepezil and **PF9601N**, simultaneously inhibiting ChE to boost cholinergic transmission and MAO to raise catecholamine levels (Bolea et al., 2013).

Although **ASS234** is a reversible inhibitor of both ChEs with micromolar affinity, it is a highly potent irreversible MAO A inhibitor, similar to clorgyline. Although the initial reversible binding parameter (K_*i*_ value of 0.4 μM) indicated that **ASS234** has a lower affinity for MAO A than clorgyline (K_*i*_ value of 0.02 μM), and this was also reflected in the higher K_*I*_ for the irreversible reaction, the full inactivation of MAO A was rapid. The crystal structure of hMAO B after inactivation by **ASS234** highlighted the formation of a covalent adduct with the flavin N5 atom which, based on the spectral changes, occurs also with the MAO A cofactor (Esteban et al., 2014). Although the *N*-benzylpiperidine moiety is not fully visible in the electron density in the crystal structure at 1.8 Å resolution, the mass determinations demonstrate that **ASS234** binds as the intact molecule to the MAO B active site, which rules out the possibility that the inhibitor may undergo degradation in the cellular context (Esteban et al., 2014).

Next, the presumed therapeutic potential of **ASS234** was evaluated following its administration to a rat model of vascular dementia based on the permanent bilateral occlusion of the common carotid arteries with experimental vascular dementia to determine its impact on brain neurotransmitter systems. In this rat model, the administration of **ASS234** for 5 days resulted in a potent and selective inhibition of MAO A activity in brain as well as a concurrent increase in concentrations of serotonin and the catecholamines, dopamine and noradrenaline (Stasiak et al., 2014). All these findings allow us to conclude that **ASS234** is able to bind to multiple targets identifying it as an interesting MTDL molecule to be considered for therapeutic development against AD.

The mechanism by which **ASS234** plays a neuroprotective role in AD pathology remains unclear. Recent evidence suggests that the Wingless-Type MMTV Integration Site (Wnt) signaling pathway is important in neuroprotection (Toledo et al., 2008), so we investigated whether **ASS234** activated the Wnt signaling pathway (del Pino et al., 2014). Total RNA was extracted from SH-SY5Y cells incubated with **ASS234** (5 μM) for 24 h and gene expression evaluated for some members of the Wnt1 class signal (Wnt1, Wnt2b, Wnt3a) which represent the “canonical” Wnt/β-catenin pathway, and for some members of the Wnt5a class signal (Wnt6, Wnt5a) which represent the “non-canonical” Wnt/PCP and Wnt/Ca^2+^ pathways. In **ASS234**-treated cells, gene expression of Wnt2b, Wnt5a, and Wnt6 was significantly increased. Ingenuity pathways analysis (IPA) identified a number of downstream genes regulated by the Wnt canonical pathways. One of these genes, PPARδ, a key gene related to neuroprotective effects against AD, was significantly increased by **ASS234** treatment. From these results, we concluded that **ASS234** induced canonical and non-canonical Wnt pathways, which presents another possible mechanism through which this compound can mediate its protective action. Knowing that the activation of Wnt signaling rescues memory loss and improves synaptic dysfunction in transgenic mice model of AD amyloid pathology, these findings indicate that **ASS234** could be a novel promising drug for AD therapy.

In order to ascertain the suitability of MTDL **ASS234** for pre-clinical studies, the obvious, preliminary and necessary “*proof of concept*” was assessed. First of all, we investigated the effect of a single-dose of **ASS234** (0.62 mg/Kg) on cognition using the scopolamine test. Not surprisingly, scopolamine significantly decreased the exploratory preference for a novel object in the retention trial. Scopolamine-induced cognitive deficit is assessed as a decreased recognition index (RI) in comparison with non-treated and vehicle control groups. After a single dose of **ASS234, a** significant increase of the RI was observed, indicating reversal of memory impairment induced by scopolamine. Thus, the scopolamine-induced amnesia was reversed by concomitant administration of **ASS234** (0.12 mM/kg) which in fact significantly improved the cognitive performance about 13.1%, suggesting that **ASS234** exerts its therapeutic effect by enhancing natural memory processes.

Some preliminary studies have been carried out to assess the amyloid plaque burden and gliosis in the cortex of the **ASS234** treated group of the transgenic AD model, APPswe/PS1ΔE9 tg mice. Daily administration of ASS234 for 16 weeks at a dose of (0.62 mg/Kg) resulted in apparent reduction in the number of neuritic plaques in the cerebral cortex and hippocampus in comparison to vehicle treated mice. Cortical plaque deposition was significantly decreased in tg mice given the **ASS234** treatment compared to tg controls. As in the cortex, the Aβ plaque load in tg mice treated with **ASS234** also decreased in the hippocampus, although statistical significance was not reached. These findings indicate that **ASS234** has a greater effect upon plaque load in the cerebral cortex than in the hippocampus of APPswe/PS1ΔE9 tg mice.

Next, since microgliosis and astrocytosis, indicative of neuroinflammation, are prominent aspects of this AD mouse model, we proceed to identify the effect of **ASS234** on neuroinflammation through the evaluation of the immunohistochemical distribution of the astrocyte marker protein GFAP and of the microglia/macrophage-specific protein iba-1. Significantly decreased GFAP and iba-1 immunostainings were observed in the cortex of the **ASS234** treated tg mice compared with that of controls, suggesting a beneficial effect of **ASS234** on neuroinflammation. All procedures with animals were carried out in accordance with European Communities Council Directive (2010/63/UE) on animal experiments under a protocol approved by the Animal Welfare Committee of the Cajal Institute (CSIC, Madrid, Spain) and by the Institutional Animal Ethics Committee of the Spain Council for Scientific Research (CSIC), adhering to the recommendations of the European Council and Spanish Department of Health for Laboratory Animals (R.D. 53/2013). A special effort was made to reduce the number of animals used in the study, and the number of animals assigned to each group was to be kept to a minimum necessary to achieve enough significance.

The promising results described above led us to assess the hepatotoxicity and metabolism of compound **ASS234**. Preliminary toxicity studies of **ASS234** along with donepezil and tacrine were performed in parallel in the human cell line HepG2. The results showed that all three compounds reduced cell viability in a concentration-dependent manner, but at very high concentrations (100 and 300 μM) **ASS234** exhibited lesser toxicity than the reference compounds, donepezil and tacrine.

## Concluding remarks

The results presented in this review strongly reinforce the suitability of MTDLs as an appropriate pharmacological approach to be used in AD therapy. Amongst all the compounds tested, MTDL **ASS234** particularly has emerged as an interesting lead compound for the design of novel MTDL with a good MAO/AChE inhibitory potency, a significant activity against amyloid aggregation, neuroprotective and anti-apoptotic properties, as well as potent antioxidant capacities, so may have a potential disease-modifying role in the treatment of AD. Given the strong correlation of neuroinflammation with amyloid burden, our results showing that **ASS234** considerably reduces both amyloid burden and inflammation in the cerebral cortex of treated tg mice underline the beneficial action of **ASS234** in slowing the progression of AD.

From the safety point of view, the affinity of compound **ASS234** for MAO A is of concern due to well known “cheese effect,” which occurs when tyramine enters the circulation and potentiates sympathetic cardiovascular activity by releasing noradrenaline, and should be considered in future developments of the molecule by analyzing tyramine potentiation and the **ASS234** MAO selectivity in the brain. In sum, **ASS234** has clearly overcome the “*proof of concept*,” and remains our most advanced anti-Alzheimer agent for pre-clinical studies targeted to find a new therapy for this devastating disease.

## Author contributions

JM and MU wrote the manuscript. GE, IB, RR, AR, RM, MC, and LI corrected the manuscript.

### Conflict of interest statement

The authors declare that the research was conducted in the absence of any commercial or financial relationships that could be construed as a potential conflict of interest.

## Abbreviations

AD: Alzheimer's Disease
AChE: acetylcholinesterase
MAO: monoamine oxidase
MTDL: multi-target directed ligand.
